# Environmental Costs of Government-Sponsored Agrarian Settlements in Brazilian Amazonia

**DOI:** 10.1371/journal.pone.0134016

**Published:** 2015-08-06

**Authors:** Maurício Schneider, Carlos A. Peres

**Affiliations:** 1 School of Environmental Sciences, University of East Anglia, Norwich, Norfolk, United Kingdom; 2 Consultoria Legislativa, Câmara dos Deputados, Brasília, DF, Brazil; Chinese Academy of Forestry, CHINA

## Abstract

Brazil has presided over the most comprehensive agrarian reform frontier colonization program on Earth, in which ~1.2 million settlers have been translocated by successive governments since the 1970’s, mostly into forested hinterlands of Brazilian Amazonia. These settlements encompass 5.3% of this ~5 million km^2^ region, but have contributed with 13.5% of all land conversion into agropastoral land uses. The Brazilian Federal Agrarian Agency (INCRA) has repeatedly claimed that deforestation in these areas largely predates the sanctioned arrival of new settlers. Here, we quantify rates of natural vegetation conversion across 1911 agrarian settlements allocated to 568 Amazonian counties and compare fire incidence and deforestation rates before and after the official occupation of settlements by migrant farmers. The timing and spatial distribution of deforestation and fires in our analysis provides irrefutable chronological and spatially explicit evidence of agropastoral conversion both inside and immediately outside agrarian settlements over the last decade. Deforestation rates are strongly related to local human population density and road access to regional markets. Agrarian settlements consistently accelerated rates of deforestation and fires, compared to neighboring areas outside settlements, but within the same counties. Relocated smallholders allocated to forest areas undoubtedly operate as pivotal agents of deforestation, and most of the forest clearance occurs in the aftermath of government-induced migration.

## Introduction

Government-sponsored rural migration schemes often export migrants to remote and sparsely settled regions, and can have global-scale economic and social consequences, and commensurately large environmental impacts. These schemes are typically motivated by national geopolitical strategies to solidify territorial claims and/or occupy remote hinterlands regardless of the resilience of native ecosystems to a sudden human population boom and associated land-use changes. This is the case of the Indonesian transmigrations into politically disenfranchised islands [1], the land reform of African hunting farms [2], and the reallocation of Amazonian wildlands to farmers since the 1970s [3]. Agrarian resettlement programs often also displace local indigenous populations from wilderness areas, such as the cases of African national parks [4] and highland forests of Laos [5].

Brazilian Amazonia has faced multiple waves of non-indigenous population incursions since it became a 16^th^-century Portuguese colony. Salati [6] describes three major historical phases of post-Colombian Amazonian colonization: the military and missionary occupation of indigenous territories (1500–1840); the systematic exploitation of high-value nontimber forest products, especially *Hevea* latex (rubber), attracting hundreds of thousands of migrants from northeastern Brazil (1840–1955); and the agrarian settlement projects, attracting primarily small farmers from southern Brazil (since 1955). The first two phases were facilitated by the vast network of natural waterways including most navigable rivers, whereas the third has been driven by extensive infrastructure investments and road building, paving the way to the typical “fish-bone” deforestation pattern that follows primary and secondary roads [7].

Since the government-induced waves of migration to Amazonian hinterlands of the 1970s, when colonists were required to deforest to demonstrate land tenure, Brazil’s policy has shifted from rapid frontier colonisation to agrarian reform schemes, a strategy to reverse the tendency of land concentration into large landholdings. Agrarian settlements are widely demanded by social organizations as a means of reducing wealth inequality and redistributing land ownership [8]. Militant landless peasantry organizations repeatedly lobby for land-reform, and several presidential terms controlled by both Social Democrats (1995–2002) and the Workers’ Party (2003-present) have responded to these demands by annually resettling ~69,000 families from their homelands since 1995 [9].

In order to reduce unemployment and rural exodus to metropolitan areas [10], successive agrarian reform plans sustained very ambitious population resettlement targets [11, 12]. The last federal plan (2003) predicted a target of 400,000 additional resettled families, 95% of which has already been executed. The latest available records indicate that, between 1995 and 2011, a total of 1,235,130 families were translocated into 8865 settlement projects, amounting to an aggregate area of 875,599 km^2^ or ~10.3% of Brazil’s vast territory (13% of those families were settled in Amazonia). Financially, this is also an extremely expensive program, with average start-up costs per family of US$12,272 [13], and this excludes open-ended options of facilitated rural credit and social benefits for many years thereafter. In fact, this is arguably the largest and most expensive modern government-sanctioned land redistribution scheme ever implemented anywhere. For example, China’s agrarian reform program involved ~210 million families but addressed land tenure rights in areas collectively occupied since the 1950s [14], a context similar to the post-Soviet land reform scheme in Russia (40 million families: [15]). The post-independence land reform in India transferred land rights across a 98,500 km^2^ area [16], and agrarian reform in the Philippines displaced over 4.08 million households, but involved a much smaller area (59,000 km^2^: [17]).

The Brazilian Forest Act (Law 4771/1965) established several constraints on land use within private properties, protecting riparian vegetation buffers and requiring mandatory vegetation set-asides of as much as 80% of each landholding. Furthermore, new regulations by the National Environmental Council (*Conselho Nacional do Meio Ambiente*—*CONAMA*) have nominally prohibited agrarian reform settlements in any forest area requiring clear-cuts (Resolutions 289/2001 and 387/2006).

Although agrarian settlement plots are allocated and supervised by Brazil’s Ministry of Agrarian Development, there is virtually no law enforcement in settlement areas, resulting in low levels of environmental compliance. In a comprehensive assessment of 4340 agrarian settlement projects created throughout the country between 1985 and 2001, only 45% and 48% retained the minimum legally-required areas of riparian buffers and forest set-asides, respectively [18], with the highest illegal timber extraction rates reported for Brazilian Amazonia. Ironically, the Brazilian Environmental Agency (*Instituto Brasileiro do Meio Ambiente e dos Recursos Naturais Renováveis—IBAMA*) repeatedly fines the federal Agrarian Agency (*Instituto Nacional de Colonização e Reforma Agrária—INCRA*) for environmental violations. Such contradictory policies across different government offices have been exposed with mandatory power by the National Auditing Bureau (*Tribunal de Contas da União*—*TCU*) [19]. Under pressure by both TCU and public prosecutors, INCRA established in 2012 the ‘Green Settlement Program’ to deal with the environmental debt of settlements, thereby avoiding new lawsuits, although typically professing that most settlement plots *had already been deforested prior to the arrival of new settlers* [20], a defensive posture that we formally evaluate here.

A number of remote sensing studies have described patterns of local to regional scale deforestation within agrarian reform settlements in Brazilian Amazonia (e.g. [7, 21, 22, 23, 24, 25, 26]). Other studies have considered agrarian settlements within a broader geographic context, but are restricted to the original forest phytogeographic boundaries of Amazonia [27, 28, 29]. Without exception, all previous studies concluded that Amazonian colonization and land reform settlements have led to higher rates of deforestation, forest fragmentation and the growth of a fish-bone pattern of land clearance that spreads away from roads to the back end of settlement plots, mirroring the orthogonal design of settler landholdings. Amazonian agrarian settlements therefore have yet to be examined as drivers of deforestation considering a before-and-after approach at the largest possible regional scale. Here, we examine deforestation rates in forest settlement areas using an 11-year time series adjusted to each settlement area including both the pre- and post-establishment phases for 300 officially recognized agrarian reform sites across the entire Brazilian Amazon. We also go beyond the strictly forested domain of Amazonia to account for both pre- and post-establishment fire incidence for 1397 settlement projects and consider patterns of natural vegetation conversion for natural forest, savannah (*cerrado*) scrubland, and grassland ecosystems for all 1911 settlement projects established prior to 2010 by the Federal government within the entire political region of Legal Amazonia.

## Methods

### Study area and data sources

The geographic data boundaries considered here follow the political definition of ‘Legal Brazilian Amazonia’, a region comprising ~5 million km^2^ (59% of Brazil’s territory) within 10 Brazilian states. This region includes primarily closed-canopy forest areas (83.1%) but also parts of the *Cerrado* (15.9%) and *Pantanal* (1.0%) open-habitat biomes. All geographic data considered here comes from the latest official sources in the executive branch of the Brazilian Federal government, covering political boundaries, settlement polygons, vegetation and infrastructure.

Boundary polygons of agrarian settlement projects were provided by *INCRA* (http://acervofundiario.incra.gov.br/i3geo) and ancillary data from the *Sistema de Informações de Projetos da Reforma Agrária* (www.incra.gov.br). Shapefiles were matched to other databases by joining the unique Agrarian settlement polygon codes. Polygons with spatial or cross-tabular ambiguities, and those >50% outside the geographic boundaries of Legal Amazonia, were excluded from the analysis. Sustainable forestry and extractive settlements (N = 252 polygons), which are special categories with strict land use control, were also excluded, and therefore only agricultural settlements were considered for analysis. Road network and road traffic data were obtained from the Ministry of Transport (www.transportes.gov.br). Population census data for 2010, annual data on the extractive production of timber (roundlogs), firewood and charcoal, and municipal county boundaries were obtained from the *Instituto Brasileiro de Geografia e Estatística* (*IBGE*; www.ibge.gov.br). Fire incidence (hot pixel) data for a 13-year period (1999–2011) were obtained from the *Sistema de Monitoramento de Queimadas por Satélites* of the Brazilian Space Agency (*INPE*; www.inpe.br/queimadas).

Land cover data for 2002 were obtained from the Ministry of Environment *PROBIO* program (http://mapas.mma.gov.br/i3geo). Deforestation data within the forest portion of Legal Amazonia were obtained from the Brazilian Space Agency *PRODES* project [30]. Vegetation conversion polygons for all *Cerrado* and *Pantanal* land areas were obtained from the *PMDBBS* project (*Projeto de Monitoramento do Desmatamento nos Biomas Brasileiros por Satélite*) of Brazil's Environmental Protection Agency (*Instituto Brasileiro do Meio Ambiente e dos Recursos Naturais Renováveis—IBAMA*; http://siscom.ibama.gov.br/monitorabiomas/index.htm). Protected area boundaries were obtained from the Ministry of Environment (http://mapas.mma.gov.br/i3geo). Finally, we examine the effects of potential agricultural value by calculating the area-weighted average level of soil fertility for each *INCRA* settlement project on the basis of a composite soil fertility map based on a 1:3,000,000-scale digital soil map of Brazilian Amazonia that was first produced in the 1970s by the Soils Division of the Brazilian Agricultural Research Agency [31], and later enhanced to generate what is now regarded as the best available soil-fertility map for the entire Brazilian Amazon [32] (G. Schroth, pers. comm.).

### Official Data and Legal Documents

Brazilian federal government land reform programs [11, 12], the Sustainable Amazon Plan [33], the Deforestation Control Plan for Legal Amazonia [34], the National Climate Change Policy [35] and laws mentioned in the paper [36, 37, 38, 39] are all available online. The same also applies to National Environmental Council’s resolutions 289/2001 and 387/2006 ([40] and http://www.mma.gov.br/port/conama/index.cfm). Official figures for projects carried out by the federal Agrarian Agency (*Instituto Nacional de Colonização e Reforma Agrária*), including those mentioned in the main text (e.g. plot occupancy and abandonment, number of settled families and environmental compliance) are available from INCRA [9, 20, 41]. Data on degraded pasturelands have been released by Ministério da Agricultura [42]. The National Auditing Bureau’s report regarding deforestation in Amazonia and INCRA’s liability can be found online [19]. Rising demand for illegal charcoal from land reform settlements was also reported in the National Congress [43] and the press [44].

There are no available georeferenced databases to distinguish private from public lands in Brazilian Amazonia, or small from large landholdings. Land titles are archived as hardcopies in outdated land registries, and local courts are entangled in prolonged judicial battles over land ownership. *INCRA* itself was given the responsibility of resolving farmland demarcation and georeferencing issues by 2005 (Law 10,267/2001) into a comprehensive georeferenced national registry (Decree 4449/2002), but only 0.58% of this target was accomplished [45]. This deadline was subsequently extended to 2010 (Decree 5570/2005) and 2022 (Decree 7620/2011), due to *INCRA*’s limited budget and human resources. It is therefore presently unfeasible to discriminate private from public lands outside settlements, and comparing settlements with their surrounding areas requires use of (1) municipal county boundaries, (2) an arbitrary buffer or (3) the entire biome. We thus selected the first option as a conservative approach to avoid any *ad hoc* buffer size. This also provides a more conservative estimate of the settlement effect size, and precludes inappropriate comparisons between relatively developed, ‘post-frontier’ municipal counties near transport infrastructure and more remote undeveloped, ‘pre-frontier’ counties that remain largely forested.

### Geoprocessing

**Fig 1:**
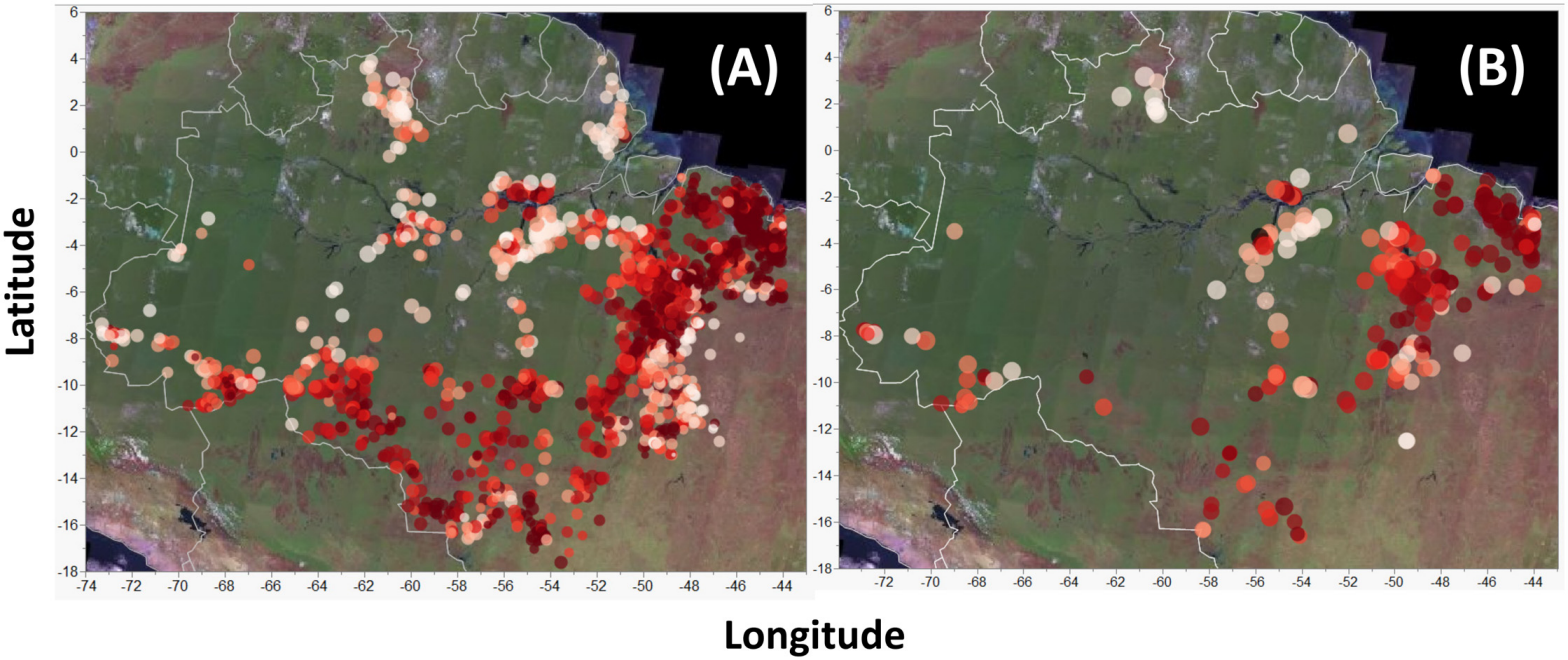
Geographic centroids of agrarian reform settlement areas. Settlements distributed throughout Brazilian Legal Amazonia were considered in terms of (A) the overall natural vegetation conversion analysis (*N* = 1911 settlements) and (B) settlements for which pre- and post-settlement deforestation data were available up to 7 years before and 11 years after the onset of agrarian settlements (*N* = 300). Sizes of circles are proportional to the log-transformed polygon areas of agrarian settlement projects, and colors indicate the proportion of land area that had been converted as of 2011 (darker colours indicate higher conversion rates).

Geoprocessing routines were performed using either ArcGIS 10 (vector files) [46] or IDRISI 17 (raster files) [47] using the South America Albers Equal Area Conic projected coordinate system and the South American 1969 datum to enable area calculations in maps straddling the boundaries of more than one UTM zone. A spatially explicit database was constructed by intersecting all 1,911 settlement polygons (Fig 1a) within Legal Amazonia with the basin-wide data sources listed above to examine the degree to which natural vegetation cover had been converted to other land uses within each settlement. A subset of 1,397 settlements established since 1988, which matched the National Space Research Agency (*Instituto Nacional de Pesquisas Espaciais—INPE*) fire monitoring program, was used to examine annual fire (hot pixel) incidence both before and after the onset of each settlement. In addition, a subset of 300 relatively recent settlements (established since 2000) located within previously forested areas was used to assess deforestation rates up to 7 years before and 11 years after the arrival of settlers (Fig 1b). These data were unavailable for other settlements because they either fell outside the original Amazonian forest biome (the area monitored by INPE) or were established prior to 2001, when INPE switched from analog to more reliable digital image classification.

A 2011 land cover raster map with 120-m pixel resolution was produced by overlaying the following shapefiles: land cover (2002); *PRODES* deforestation data within forest regions (2011); and the latest available data for the *cerrado* (2010) and *pantanal* (2009) phytogeographic boundaries. Our maps distinguished five land cover classes: forest, cerrado/grassland, bare ground (later omitted from our figures due to its negligible extent), water, and agropastoral conversion of natural vegetation. Original *PROBIO* maps distinguished 41, 36, and 29 land cover classes for the forest, *pantanal*, and *cerrado* biomes within Legal Amazonia, respectively, but this level of disaggregation proved to be unnecessary in this study. *PRODES* deforestation polygons within Amazonia were coalesced into the same class to calculate the total land conversion area, but we considered the year of deforestation for each polygon both before and after the onset of occupation of each settlement. However, deforestation areas under cloud pixels (for which the deforestation year was unknown) were excluded from any pre- vs post-settlement analyses. We considered both the *PROBIO* map (2002) and the *PMDBBS* vegetation conversion polygons (2002 to 2010), when assessing natural vegetation loss within non-forest (*cerrado* and *pantanal*) biomes. Annual data on conversion into agropastoral land-uses were unavailable for these biomes, thereby restricting our before-and-after assessments of vegetation conversion to the forest domain accounting for 83.1% of the Brazilian Legal Amazonia region.

Human population density (HPD) within settlement projects was estimated in terms of the number of smallholder families reported to have settled per km^2^. We also estimated the HPD outside each settlement project by creating a 10-km external buffer and intersecting all georeferenced households from the latest (2010) *IBGE* national census. We calculated the Euclidian distance from each settlement project to the nearest major paved and unpaved road (managed by a state or federal agency) for which estimates of traffic intensity are available in terms of cumulative number of heavy vehicles (cargo or passengers) per day. Annual extractive production of timber, charcoal and firewood within settlements was estimated using the area-weighted average of the municipal county scale data by overlapping the *IBGE* data for each resource type within county boundaries onto the settlement polygons.

### Data analysis

The relative influence of environmental predictors on the conversion rate of natural vegetation cover (including both forest and *cerrado*) within each settlement polygon was examined using generalized linear models (GLMs). We attempted to control for high levels of variable inter-dependence by performing a Pearson correlation matrix, but none of the explanatory variables were intercorrelated by |r| > 0.70. Our main response variables were (1) cumulative conversion rates of any natural vegetation for all 1,911 settlements, as of 2011; and (2) ΔDeforestation, defined as the mean difference in annual deforestation rates *before* (until year_–1_) and *after* (since year_+1_) the creation of any given settlement at year 0 (year_0_) for the relatively recent 300 settlement areas for which both pre- and post-settlement deforestation data were available. Our predictors included several key variables describing the biophysical setting, physical accessibility, level of soil fertility, human population density, and the socioeconomic and historical profile of each settlement area, such as settlement age, which were extracted for each polygon (see variable descriptions in Table 1). GLMs modelling all settlements used a binomial error structure with a logit link to investigate the proportion of pixels representing natural vegetation loss relative to the total number of pixels contained by each settlement polygon. We also fitted GLMs to the total number of pixels in each settlement area where the original vegetation had been converted, assuming a Poisson distribution and a log link, and treating settlement size (total number of pixels) as an offset variable. However, there were no differences in variable effect sizes between these two approaches, given the large number of pixels per settlement. Δ Deforestation was modelled as a continuous variable using a Gaussian error structure. We ran all GLMs both with and without mean human population density (HPD) outside settlement areas, as this variable was only available for ~2010, although HPD_2010_ was likely to be spatially correlated with that in earlier annual periods.

**Table 1:** Explanatory variables used in deforestation and land-use conversion models in this study.

Explanatory variables	Variable description and units
Total settlement area	Area (ha) of each agrarian settlement project (log_10_ x)
Internal human population density	Number of smallholder families settled per unit area—families km^-2^ (log_10_ x)
Settlement occupation capacity shortfall	Density differential between the predicted number of families that could be settled and the number of families that were actually settled per unit area (capacity—settled) km^-2^, log_10_ x + 1).
Age of settlement	Time (years) since the official establishment of the settlement project
Human population density immediately outside settlements	Density of households (per km^2^) within a 10-km buffer area surrounding each settlement polygon (log_10_ x)
Soil fertility	Area-based weighted mean level of composite soil fertility within settlement areas
Pre-settlement deforestation rate	Proportional forest loss (%) one year prior to the arrival of settlers
Distance to nearest major road	Euclidean distance (km) to the nearest major paved and unpaved road (log_10_ x).
Traffic intensity of nearest major road	Overall traffic intensity defined as the flux of cumulative heavy cargo and passenger vehicles (heavy vehicles/day)

To examine differences in annual fire incidence and vegetation conversion rates (of forest and/or natural cerrado) within and outside settlement areas throughout the entire Legal Amazon region, we used paired t-tests [48]. These pairwise comparisons included the physically demarcated settlement polygons and all areas outside settlements but within municipal county boundaries, thereby controlling for the wide variation in geographic and socioeconomic contexts of frontier expansion that affect deforestation rates [49]. We used Fisher's exact tests [50] to compare deforestation rates within settlement polygons with any area outside the settlement but within the same municipal county. Of all 771 Amazonian counties, however, only those containing settlement projects (*N* = 568) were considered. All statistical analyses were performed using R [51].

## Results

Migrant families resettled into Amazonian agrarian reform plots set aside by INCRA currently represent 18.0% of the total rural population of Legal Amazonia of ~6.71 million people, a disproportionately high aggregate population density in that agrarian settlement plots account for 267,092 km^2^ or only 5.3% of the overall region (Table 2). Hence, INCRA settlements (~4.52 persons km^–2^) support 3.9-fold the average human population density of all rural Amazonian areas outside settlements (~1.16 persons km^–2^). Assuming a mean resettled family size of six persons, we also estimate that the internal HPD within any given settlement is on average 75.3 times (CI_95%_ = 55.8–94.9) greater than that the external HPD in a 10-km buffer area outside that settlement.

**Table 2:** Summary of land cover, fire incidence and rural population size for the entire Legal Amazon administrative region, distinguishing areas within and outside INCRA agrarian reform settlements.

	Legal Amazonia		Agrarian settlements		Public and private land outside settlements	
Total area (km^2^)	5,030,583		267,092	5.3%	4,763,491	94.7%
Rural households (2010 census data, INCRA 2012)	1,965,216		353,243	18.0%	1,611,973	82.0%
Rural population (2010 IBGE census)	6,710,666		1,206,227		5,504,439	
Fire incidence (annual mean hot pixels km^-2^)	0.020		0.054		0.018	
Land cover, km^2^ (and %)						
Forest	3,116,448	62.0%	105,794	39.9%	3,010,653	64.7%
Cerrado/Pantanal	710,788	14.1%	12,405	4.7%	698,384	15.0%
Agropastoral conversion	1,092,211	21.7%	146,937	55.4%	945,274	20.3%
Total land area (excluding water)	4,919,447		265,136		4,654,311	
Water	111,136	2.2%	1,956	0.7%	109,180	2.3%

**Fig 2:**
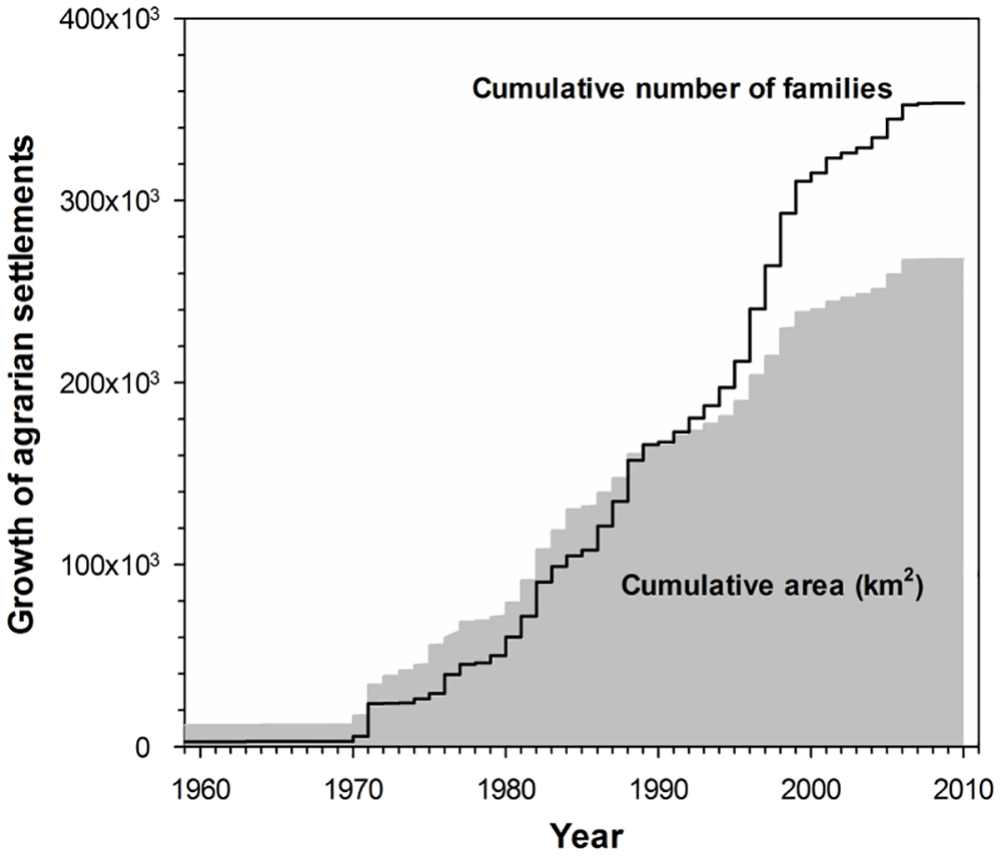
Settlement growth since the 1960s. Cumulative growth over time in both the total area allocated to INCRA settlement areas (shaded area) and the number of settler families (solid line) occupying those settlements throughout the Brazilian Legal Amazon administrative region.

The total land area set aside for agrarian settlements grew steadily since the early 1970s, with a mean area of 6394 ± 4776 km^2^ added every year. There was also a steady growth in the number of families migrating into planned settlements each year (mean ± SD = 8762 ± 7677 families yr^-1^ from 1970 to 2010), but this was greatly elevated during the 1990s, with historical peaks of over 28,000 families yr^-1^ resettled in 1996 and 1998. By 1990, the cumulative growth in settler in-migration outpaced that of the cumulative area they occupied, indicating that smaller plots were being allocated to each migrant family (Fig 2). Indeed, the average plot area decreased seven-fold from an annual mean of 392.6 ± 1,005.2 ha in 1970–1989 before 1990 to only 55.9 ± 20.5 ha since 1990. As we shall see, this increase in internal population density has a significant effect on deforestation rates within settlements.

### County-scale deforestation

**Fig 3:**
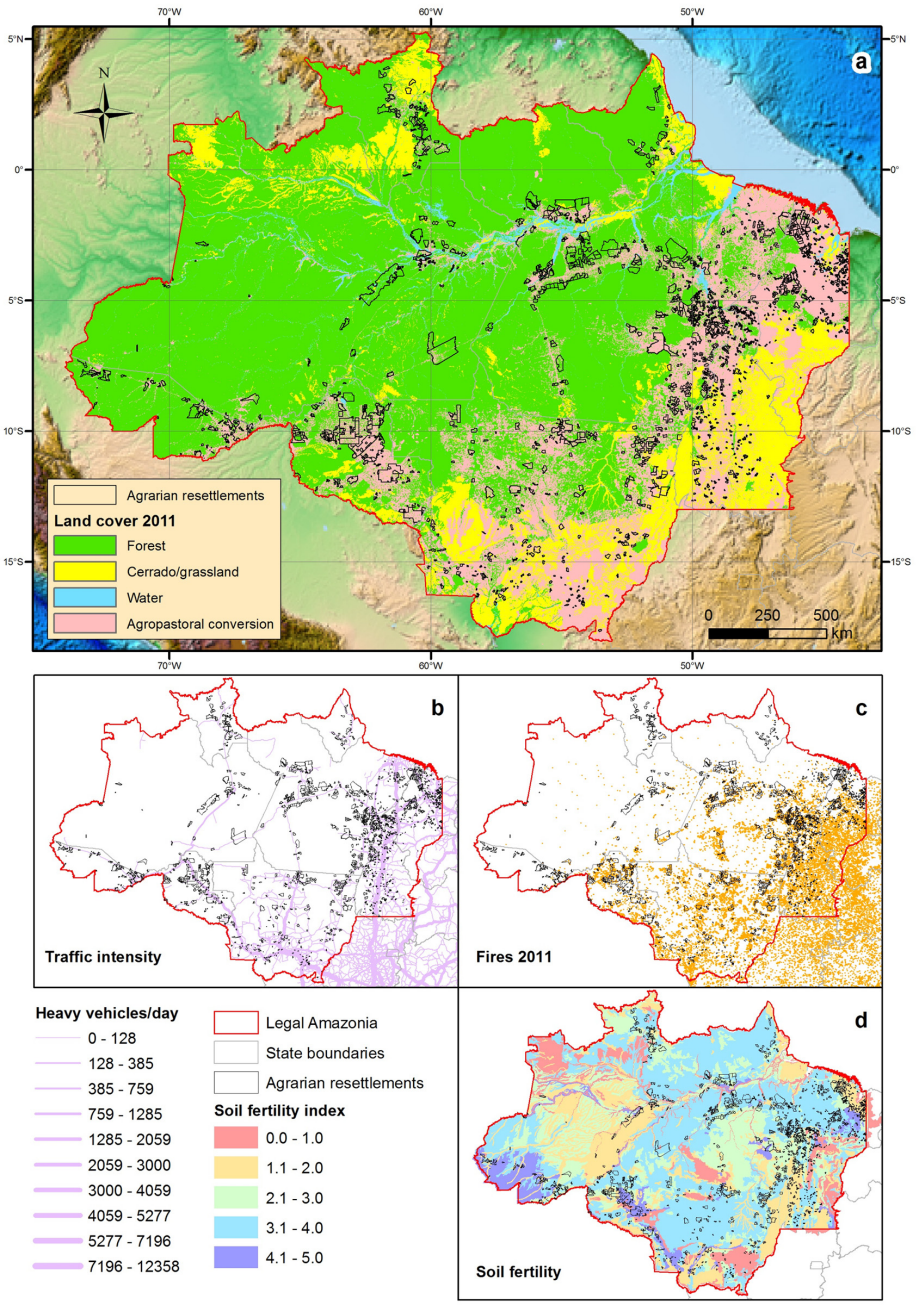
Agrarian reform settlement areas within Brazilian Legal Amazonia. Main map showing (a) land cover as of 2011 and state administrative boundaries (topographic map background from [52]). Smaller maps show (b) the level of traffic intensity along major roads, expressed in terms of the number of heavy cargo and passenger vehicles per day, (c) the spatial distribution of fires (hot pixels) in 2011, and (d) major classes of agricultural soil fertility throughout the region.

**Fig 4:**
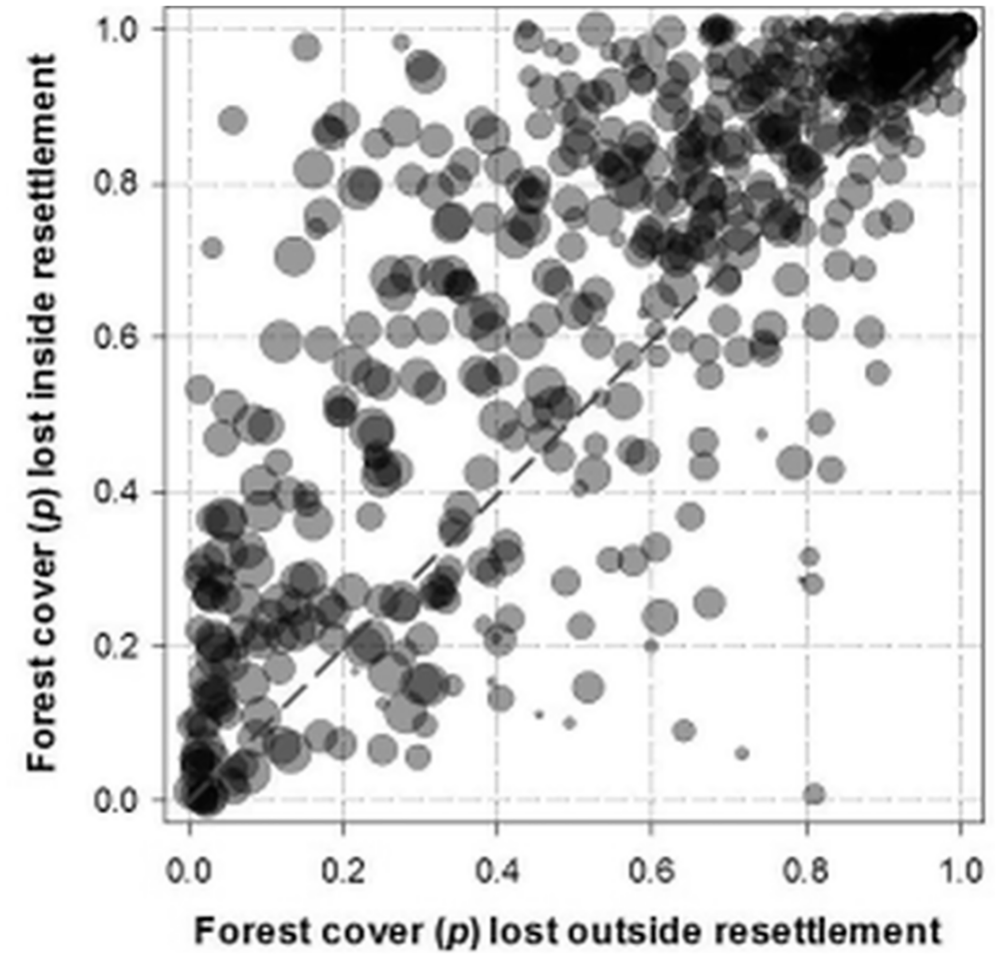
Proportion of vegetation loss inside and outside settlements. Municipal county scale proportion of natural vegetation cover lost both inside and outside the boundaries of INCRA agrarian settlements for the 568 counties of Legal Amazonia containing settlements. Sizes of circles are proportional to the size (log_10_ x) of counties. All circles above the dashed diagonal line indicate counties that have lost a higher proportion of forest cover within settlement polygons than areas outside.

Over half of the total area (55.4%) allocated to agrarian reform settlements has already been converted into anthropogenic land-uses. Agrarian settlements in Legal Amazonia have been primarily established near major roads to ensure access to regional and national markets (mean distance to roads = 13.0 ± 21.2 km, Fig 3b). However, these settlements range widely in geographic and landscape contexts from those dominated by the agricultural frontiers of eastern and southern Amazonia to pristine forest regions with little accessibility. We therefore controlled for landscape context by comparing forest and non-forest conversion rates within and outside settlement polygons for 568 of the 775 municipal counties of Brazilian Amazonia containing agrarian settlements. Within these counties, overall conversion rates of natural vegetation represent 56.8% of the total agrarian settlement area compared to only 24.3% of the total area outside settlements (Fisher's exact test, *p*<2.2^−16^). County-scale deforestation rates within and outside settlement projects was highly correlated (r = 0.729, p < 0.001), but on average 2.14-fold higher (95% CI = 1.83–2.45) within settlement projects than in areas outside (paired t-test, *t* = 10.37, p<0.001). The proportion of natural vegetation cover remaining within settlements as of 2011 was lower than that elsewhere in the same county for 73.9% (420 of 568) of the counties containing settlements (Fig 4). Heavily deforested counties often had higher deforestation rates both within and outside settlements, whereas highly forested counties lost proportionally much more forest cover inside settlements than in areas outside. This relationship is mediated by county size, since larger counties exhibit much lower deforestation rates (r = –0.650, *N* = 568). This is explained by the fact that county area in Amazonia explains virtually the entire variation (R^2^ = 98%) in county-scale human population density, so that large, sparsely-settled counties typically exhibit low deforestation rates as a percentage of area, but what deforestation does occur in those counties tends to occur inside INCRA settlement boundaries.

The conversion rate of any pre-existing natural vegetation into agropastoral land-uses, was 2.5-fold higher inside settlement polygons than in areas within the same municipal county but outside settlements (*t* = 10.19, *p* < 0.001). Likewise, the overall incidence of fire (hot pixels) was 3.1-fold greater inside settlements, compared to same-county areas outside (*t* = –5.06, *p* < 0.001).

**Fig 5:**
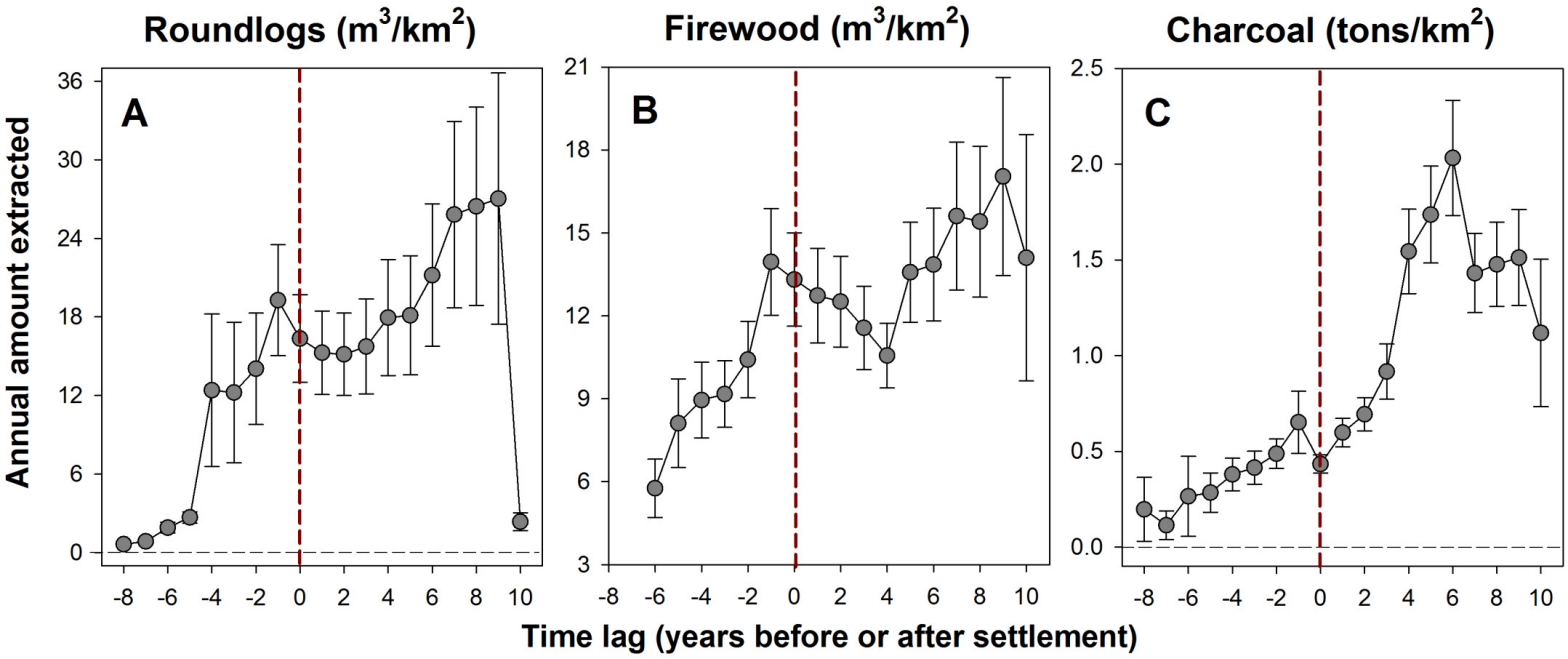
Extractive production before and after settlements. Area-weighted average extractive production of (A) raw timber (roundlogs), (B) firewood, and (C) charcoal for counties containing agrarian settlements, estimated on the basis of municipal county scale data overlapping all settlements for which annual offtake data from IBGE (2000–2010) were available (*N* = 424) for up to 8 years before (yr_‒8_) and 12 years after (yr_+12_) the formal establishment of settlements. Error bars indicate standard errors.

Municipal counties containing settlements also showed marked differences in reported annual extractive rates of forest products (roundlogs, firewood and charcoal) before and after the formal arrival of settlers. Roundlog production for INCRA-settled counties began to increase up to 5 years before the formal onset of settlements, and continued to grow at a slower pace for up to 9 years thereafter until it suddenly crashed, most likely due to local depletion of high-value timber stocks that are highgraded by loggers (Fig 5a). This resulted in significantly different linear regression slopes before and after the onset of settlements (*t* = 5.44, *p*<0.001). Firewood production also began to increase steadily well before the formal occupation of settlements (difference in slopes, *t* = 5.39, *p*<0.001; Fig 5b), whereas charcoal production showed a transition from modest to much higher increases before and after settlements, followed by a sharp collapse in yields 6 years after the formal establishment of settlements (difference in slopes, *t* = 11.02, *p*<0.001; Fig 5c).

**Fig 6:**
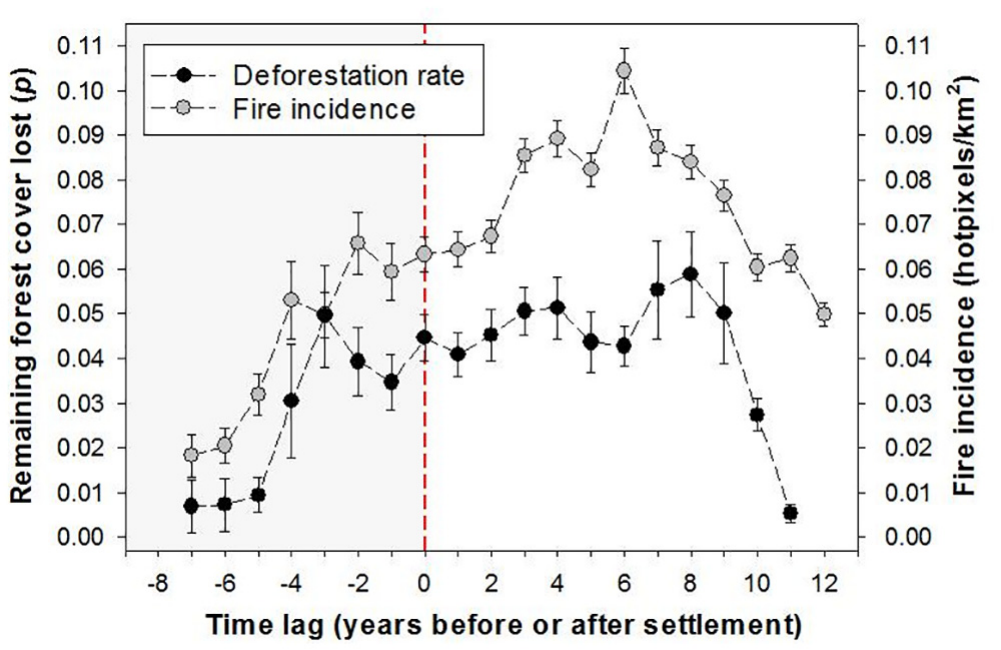
Deforestation and fire incidence before and after settlements. Variation in annual deforestation and fire incidence rates within agrarian settlements within Brazilian Amazonia. Relative annual deforestation rate is expressed as the proportion of additional loss in the forest cover remaining from the previous year. Solid and shaded circles represent mean deforestation rates and mean fire incidence calculated from 300 and 1397 settlement areas, respectively, for which reliable data are available. Error bars indicate standard errors.

**Fig 7:**
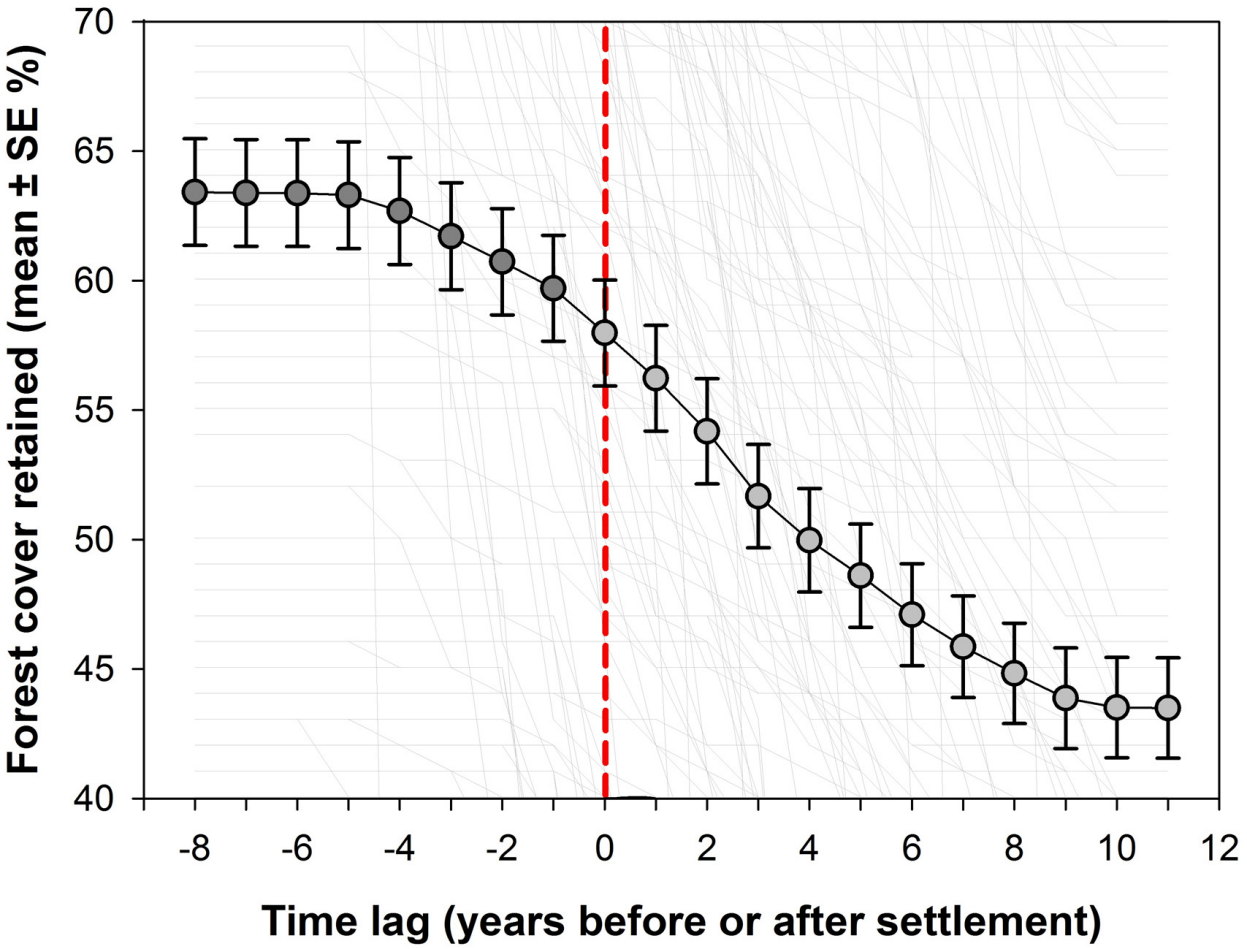
Forest cover retention before and after settlements. Mean annual deforestation within 300 agrarian settlement areas across Brazilian Amazonia, up to 7 years before (yr_‒7_) and 11 years after (yr_+11_) their formal establishment. Vertical dashed line indicates the official recognition of any given settlement at year zero (yr_0_). Forest loss prior to year zero was often associated with agricultural activity by land squatters. Error bars indicate standard errors.

Although agrarian settlement areas established after 2000 had retained less than two thirds of their forest cover immediately prior to establishment (mean ± SD = 59.7 ± 35.3%), relative deforestation (defined as the proportion of additional loss in the forest cover remaining from the previous year) increased markedly, reaching a peak 4 years after the onset of settlements. As expected from typical slash-and-burn agriculture (cutting down and burning the forest), the incidence of fires to clear forest biomass also increased dramatically following deforestation (Fig 6). This was followed by a sharp decrease in both deforestation rates and density of fire pixels as the total amount of remaining forest cover declined to an average of 43.5% (SD = 33.5%) some 10–11 years after the arrival of settlers (Fig 7).

### Drivers of deforestation within settlement areas

**Fig 8:**
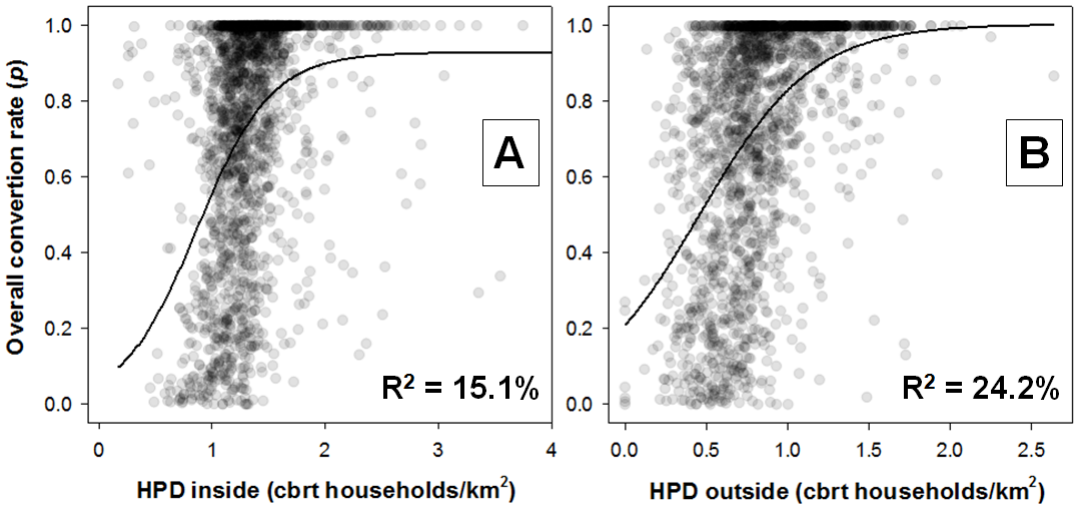
Human population density *vs* land conversion. Relationship between human population density (HPD), expressed in terms of the cubic root of households/km^2^, both (A) within and (B) outside INCRA settlement project areas and the proportion of natural forest, *cerrado* and grasslands in these areas that has been converted to any agropastoral land-use. R^2^-values, which represent best-fit lines from 3-parameter sigmoidal functions, indicate that landscape-scale HPD has played a greater role in driving historical vegetation conversion rates within settlement areas than the density of families of small farmers who were actually relocated into those settlements.

**Fig 9:**
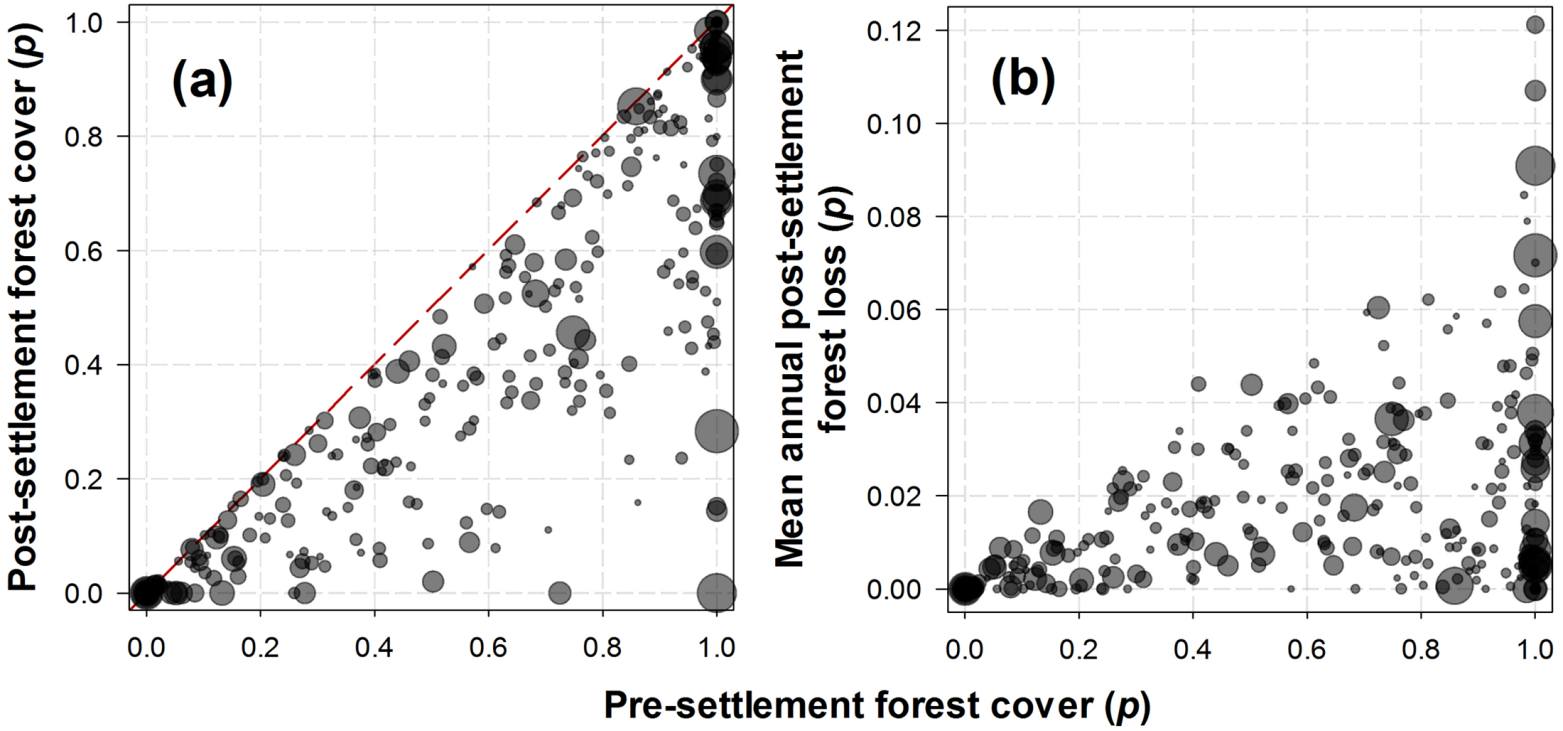
Proportion of forest cover and human population density. Pre- and post-settlement (a) forest cover and (b) mean post-settlement deforestation rates within agrarian reform settlement areas across the Brazilian Amazonia. Circle size represents the landscape-scale human population density (log_10_ households km^-2^) within a 10-km buffer area outside settlements.

GLMs clearly highlight the importance of settlement size and within-settlement human population density as major drivers of land-use change (Table 3, Fig 8). The proportion of forest and/or *cerrado* was drastically reduced within agrarian reform areas embedded within densely-settled landscapes, even if settlement polygons had been largely intact prior to occupation (Fig 9a). The overall magnitude of landscape-scale economic activity and access to regional and national markets via major roads under heavy usage—defined in terms of both distance from nearest roads and their overall usage by heavy vehicles—were strong determinants of cumulative vegetation loss. Area-weighted soil fertility within settlements was also a strong, secondary predictor of conversion rates to agropastoral land uses, with smaller, densely-settled settlements on more fertile soils being converted faster than larger, sparsely-settled areas of lower agricultural value. Surprisingly, older settlements retained a proportionately greater natural vegetation cover, presumably because many of them were allocated to large landholdings or had low agricultural value, although this time-lag effect was relatively weak compared to others discussed here.

**Table 3:** GLM model results (slope coefficients and associated ± SE) of predictors of cumulative vegetation (forest and cerrado) conversion rate as of 2011 within agrarian settlement areas across the Brazilian Legal Amazon region (N = 1,911); and mean difference in annual deforestation rate *before* (until year_–1_) and *after* (since year_+1_) the creation of settlements (*N* = 300).

		Vegetation conversion rate		Δ Post:Pre deforestation^a^	
Model components	Units	Global	Best^b^	Global	Best^b^
Total settlement area	Hectares (log_10_ x)	**–0.355 ± 0.019** **	**–0.346 ± 0.115** **	**–0.373 ± 0.180** *	**–0.380 ± 0.122** **
Human population density of settlement	Families km^-2^ (log_10_ x)	**1.202 ± 0.212** ***	**1.149 ± 0.209** ***	0.035 ± 0.279	
Settlement occupation capacity shortfall	(predicted family capacity—number of families settled) km^-2^ (log_10_ x + 1)	–0.028 ± 0.363		–1.115 ± 3.307	
Age of settlement ^c^	Years since establishment (sqrt x)	0.013 ± 0.050		NA	NA
Soil fertility	Weighted mean soil fertility index	**0.076 ± 0.052** ^†^		**0.250 ± 0.065** ***	**0.254 ± 0.063** ***
Pre-settlement forest cover ^d^	Forest cover (%) in settlement polygon remaining in the year prior to settlement establishment (yr_–1_)	NA	NA	**1.478 ± 0.202** ***	**1.523 ± 0.197** ***
Distance to nearest road	km (log_10_ x + 1)	**–0.143 ± 0.032** ***	**–0.141 ± 0.032** ***	0.049 ± 0.046	
Traffic intensity of nearest road	Mean flow rate of heavy vehicles (trucks and buses) per day (log_10_ x)	**0.271 ± 0.049** ***	**0.272 ± 0.050** ***	**0.214 ± 0.071** ***	**0.223 ± 0.070** ***
Model deviance explained (%)		15.67	15.48	22.20	21.88
Model AICc		1994.4	1995.8	962.6	957.8

^a^ Refers to the difference in mean annual rate of forest loss before (t ≤ yr_–1_) and after (t ≥ yr_+1_) the onset of agrarian settlements (see text). Age of settlement was therefore omitted from these models.

^b^ Most parsimonious model selected based on multiple comparisons of AIC values.

Significance levels:

^†^ < 0.10,

* < 0.05,

** < 0.01,

*** < 0.001.

^c^ Omitted from Δ Deforestation models because our before-and-after deforestation rate already considers the settlement time trajectory;

^d^ Omitted from vegetation conversion rate models due to data unavailability for all but the 300 settlement polygons for which Δ Deforestation models were performed.

The ‘best model’ explaining post-settlement deforestation rates (for the 300 settlements for which we had detailed pre- and post-settlement annualized deforestation data) included only the proportion of forest cover that had been retained at the time of settlement occupation, the total settlement area, the traffic intensity of nearby roads, and our composite index of soil fertility.

## Discussion

Our analysis unambiguously shows that government-incentivized migrations of small farmers into the Amazon greatly accelerate the conversion rate of natural ecosystems into agricultural or highly degraded areas. Other smaller-scale studies may have shown high deforestation rates in agrarian settlements, but this is the first analysis considering the fate of all INCRA settlements throughout Brazil’s entire Legal Amazon region, including natural forest, *cerrado* and grassland biomes. We also present new data on annual deforestation rates and fire incidence over 9 years before and 12 years after the onset of settlements to examine whether deforestation within settlements largely predates the arrival of settlers, as often claimed by INCRA’s reports, or is primarily driven by newly settled farmers. According to INCRA’s official discourse, there has been a radical shift in environmental policy in which settlements since 2000 have successfully avoided the predatory land-use practices that were typical of earlier settlements for which they were so heavily criticized [20]. Contrary to these claims, however, our analysis of annual deforestation trends clearly shows that settlements established between 2000 and 2010 continue to accelerate deforestation rates, at an average rate of 4.4% (SD ± 10.2%) per year.

**Fig 10:**
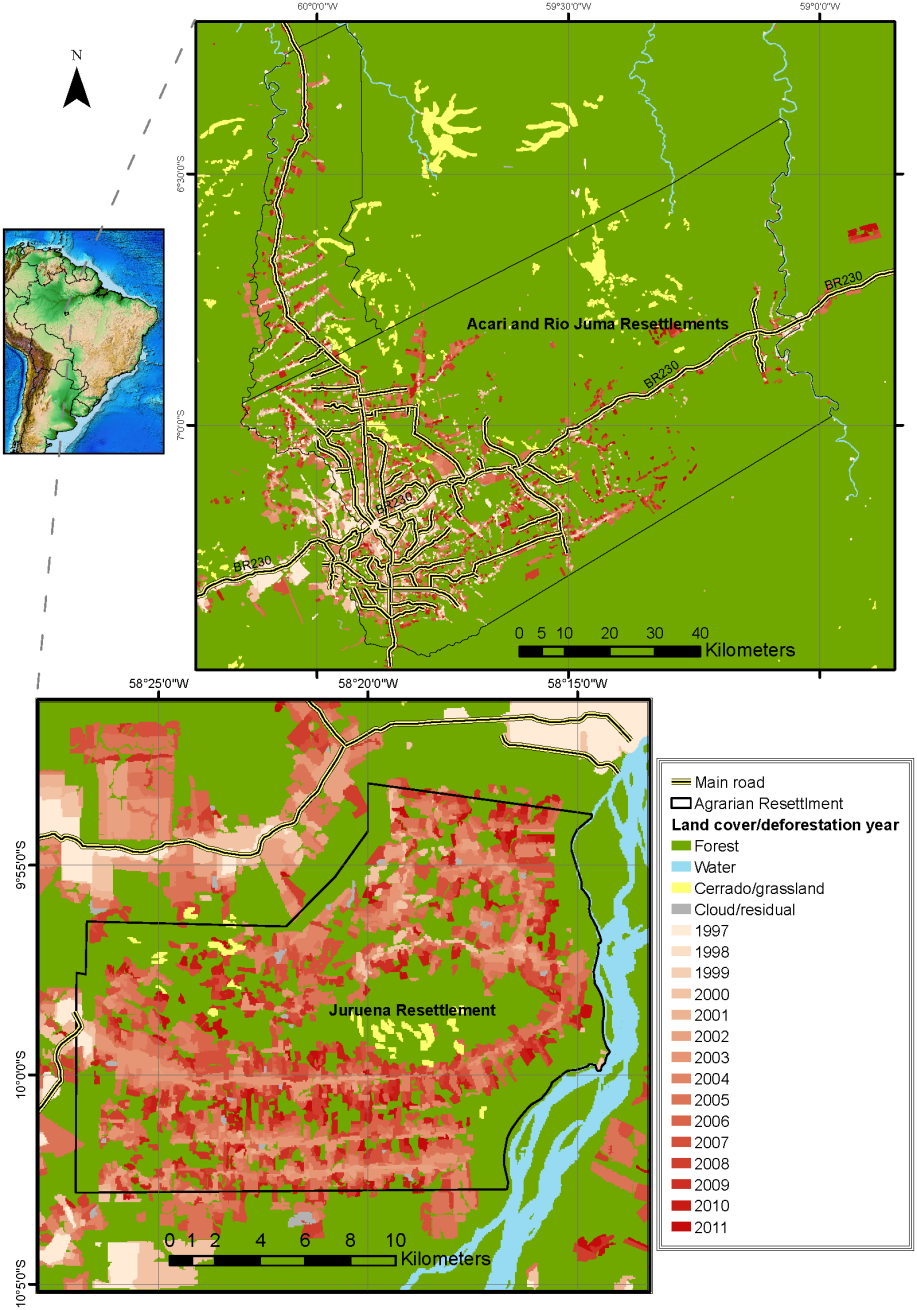
Annual deforestation polygons in selected settlements. Examples of land cover change within three INCRA settlements of the Brazilian Amazon. The Rio Juma and Acari Settlements (top) were created in 1981 and 1982, respectively, far from the southern Amazonian agricultural frontier, whereas the more recent Juruena Settlement (created in 1997) lies in a vibrant deforestation hotspot of the northern state of Mato Grosso. These settlement polygons had experienced very little loss in forest cover prior to the arrival of settlers, as indicated by the color-coded deforestation chronosequence (where darker colors indicate more recent deforestation).

Fire pixel density and deforestation rates inside agrarian settlement boundaries were roughly three times higher than those immediately outside. INCRA-style settlements have either accelerated forest loss within municipal counties that had already been encroached by agropastoral conversion or catalyzed the onset of deforestation within relatively remote and largely intact counties. This is illustrated by a deforestation time-series at two settlements within markedly contrasting landscape contexts between the expanding agricultural frontiers penetrating the eastern and southern fringes of Amazonia and those elsewhere (Fig 10). The Juruena Settlement was established in 1997 in Cotriguaçú (northern Mato Grosso), one of the top-ranking deforestation counties in Amazonia. Although recent deforestation occurred primarily within the settlement boundaries, post-settlement deforestation clearly spread rapidly by contagion along neighboring roads. In contrast, the Rio Juma and Acari Settlements (created in 1981 and 1982, respectively) lie in the central state of Amazonas, which still retains 97.3% of its original forest cover. In this relatively remote region, there is little evidence of forest loss, unless this occurs along new roads inside agrarian settlements in a contagious pattern observed throughout the Amazon [53].

Settlements located in densely populated counties are typically embedded within socioeconomically effervescent landscapes dominated by a dynamic rural private sector. Economic demands by newly settled households are therefore aggravated by external pressures, such as timber extraction, charcoal production, and leasing of previously logged deforestation-prone landholdings [54, 55]. Although physical proximity to roads and large human populations have been identified as key drivers of deforestation [56], we find that the intensity of road traffic is a more important correlate of deforestation than road accessibility per se. As our measure of soil fertility shows, this was particularly true for agrarian settlements allocated to nutrient-rich soils [57], which presumably attracted greater agricultural conversion and faster landholding consolidation.

A breakdown in deforestation trajectories eight years following settlement occupation mirrors the abrupt decline in extractive output of timber forest products estimated for each settlement project (Fig 6). Annual roundlog extraction, which had already been escalating well before the official establishment of new settlements—rather than accelerating during post-settlement years—grew steadily for about a decade prior to a sudden collapse typical of most boom-and-bust extractive industries. Firewood is more important as an everyday fuel source in household subsistence than as a trade commodity, which explains the absence of an exponential increase typical of post-settlement commercial production. Charcoal production, on the other hand, rapidly increased over the first six post-settlement years. Charcoal has become a critical trade commodity in the eastern Amazonian metallurgical sector (in converting iron ore into pig iron), fuelling a voracious appetite for cheap, and illegally produced charcoal [58]. Although illegal charcoal production from old-growth forests within agrarian settlements had been documented (e.g. [43, 59, 60]), our study clearly uncovers a pattern of relentless erosion of forest resources associated with government-induced settlements.

In addition, the history of deforestation prior to *de jure* occupation by new settlers cannot be regarded as spatially independent from the nature of landholding allocation to agrarian reform by INCRA. The standard tactics of several organizations promoting landholding take-over by landless squatters in Brazil is to initially occupy ‘undeveloped’ private or public forestlands that had been preselected for redistribution, and only later demand expropriation and official recognition of settler land deeds [3, 61, 62]. Therefore, for several years leading up to the formal demarcation of a new settlement, there is often a *de facto* occupation resulting in significant forest loss, which begins to escalate ~4 years prior to the official decree of INCRA settlement status (Fig 9). In fact, organized land squatters prefer targeting forestlands where they can access timber resources for both house construction and trade with illegal loggers, which is critical in capitalizing small farmers prior to agricultural investments [8, 54].

Although some authors argue that Amazonian deforestation may be primarily driven by medium- and large-holders [63], deforestation figures are cumulative, and county-scale agrarian structure data only capture a given moment in time. In post-frontier regions, wealthier farmers acquire deforested land from smallholders, many of whom are agrarian reform settlers (e.g. [64]), resulting in a misleading spatial correlation between predominantly large farms and deforestation. When we consider georeferenced farm boundaries, rather than county-scale proportions of large/medium/small farms, landholding size becomes a key determinant of property-scale deforestation rates, whereby small properties constrained by small economies of scale can only retain proportionally less forest cover [65], [66]. This is consistent with the gradual transition of deforestation polygons from large (>1,000 ha) to small patches (<50ha) over the last decade [67]. Settlement polygon area, which predicted 40.5% of the variation in within-settlement household density, was a significant negative predictor of both the overall proportion of vegetation loss and post-settlement deforestation rates of INCRA settlements (Table 3, see also Fig 8). The land tenure structure of these settlements, which in the last two decades have allocated gradually smaller lots to each family, further induces a more intensive land-use pattern, coercing new settlers to deforest a proportionally larger landholding area. In fact, household density within settlements was the most important predictor of the proportion of settlement areas converted to other land-uses. The typical response of families resettled into small farms, which typically lack various economies of scale, is to either increase deforestation or sell out their lots to larger farms [23]. Both of these economic strategies are in fact complementary, given that traditional slash-and-burn agriculture demands at least 25 ha to sustain a family of five even in relatively fertile portions of Amazonia [7]. Furthermore, plots smaller than four “fiscal units” (corresponding to an average of 304 ha in Legal Amazonia) are defined as a smallholding (Law 8629/1993). Complying with the minimum 80% forest set-aside within a smallholding would leave a typical resettled family with barely enough farmland to meet its own subsistence needs. Strict (albeit obviously mandatory) application of the law would increase the already high family turnover and giving-up rates of newly settled farmers within settlement projects (mean ≈ 30%, but up to 84% of all settlers in extreme cases) whereby many families move on to new areas only a few years after arriving in a given settlement [68, 69]. The agrarian agency reclaims ~14,000 plots year^-1^ due to abandonment or illegal occupancy [41]. Yet this relentless wave of new settlements continues apace despite the high environmental costs, and high monetary costs to the national treasury. In fact, agrarian settlement projects in northern Brazil are effectively small enterprises [70] that can rarely afford to comply with environmental law.

The average start-up cost per family in northern Brazil has been estimated at ~US$14,800 [13], but this takes no account of social benefits, debt pardoning, and an open-ended stream of rural credit paid annually to subsidize each family in overcoming the economic inefficiencies inherent to agriculture far removed from adequate infrastructure and consumer markets. This results in an average family income from both agriculture and social benefits paid to agrarian reform settlers in Amazonian states ranging from 0.75 to 1.47 times the national minimum wage of ~US$80 month^-1^ in 2003 [10]. In fact, from a strictly macroeconomic and environmental perspective, it would probably be cheaper and safer to subsidize landless peasants *not to migrate* to new settlements in pristine forestlands [71]. This is particularly true if INCRA continues to select ‘economically idle’ primary forest areas for agrarian reform. Reclaiming severely degraded pastures in Amazonia costs ~US$900 ha^-1^ in agricultural inputs, but this can be repaid to a break-even point within 4.5 years, depending on the land-use option, including croplands (e.g. soybean, rice, maize) or renovated cattle pastures [72]. Although reclaiming low-productivity pastureland for new settlement areas would be initially expensive, this would both boost income of resettled families and lead to much lower overall environmental impacts.

We acknowledge, however, that in November 2012 INCRA launched the ‘Green Settlement Program’, to control deforestation, reduce environmental debt, and promote ‘best practices’ inside land-reform projects [20]. However, it is not yet possible to assess the environmental performance of this program at such an early stage.

## Conclusion

The current political agenda of the Agrarian Reform Office is still in direct conflict with Brazilian environmental laws and regulations enshrined by explicit environmental policy of the Federal Executive, reiterated in the Sustainable Amazon Plan [39], the Deforestation Control Plan for Legal Amazonia [34], and the National Climate Change Policy [35], all of which seek to curb or prevent illegal deforestation. Moreover, INCRA settlement projects are often in clear contravention of official environmental policy in lacking the basic mandatory environmental license required of any other regional development project [73].

There are also wider fundamental land-use conflicts in haphazard frontier expansion. For example, we overlaid all agrarian settlement polygons with the boundaries of all Amazonian strictly-protected and sustainable-use protected areas, amounting to a total overlapping area >798,600 ha of conservation units managed by both state and federal agencies (even excluding Environmental Protection Areas, which tolerate agricultural conversion). This overlapping area with diametrically opposite objectives undermines both state and federal protected areas, and exposes severe contradictions between the federal agrarian reform program and environmental policies led by the federal Ministry of Environment and state environmental offices. The physical demarcation of agrarian settlements inside protected areas often results in even wider judicial consequences since illicit logging and deforestation by settlers will inevitably take place within legally protected areas (Law 9605/1998), and land claims for agriculture have proved to be a major cause of downgrading, downsizing and degazettement of protected areas in Brazil [74].

INCRA’s settlement program has been carried out without proper environmental licensing and in clear contempt of constraints established by the Brazilian Forest Act (Law 4771/1965), which prescribed forest set-asides as buffer riparian zones, other *Permanent Preservation Areas*, and mandatory *Legal Reserves* amounting to as much as 80%, 35% and 20% of the landholding size in forest, *cerrado* and grassland biomes, respectively. However, this Act was revoked in 2012 by Law 12,651, and despite maintaining these proportional set-asides, it included environmental amnesty provisions favouring previously deforested landholdings, which will condone not only violations by private landowners, but also the federal agrarian agency and its resettled beneficiaries.

Considering that 55.4% of the natural vegetation cover of all 1,911 settlements has already been converted into other land uses, and that only 20% of the 1,701 once-forested settlement areas could be deforested, the outstanding environmental liability of the official agrarian reform program in Brazilian Amazonia approaches one third of the total land area of all settlements (≈ 26.5 million ha). However, this fails to consider other illegal activities such as hunting, timber extraction, burning, and conversion and/or degradation of riparian *Permanent Preservation Areas* which typically accompany population in-migration and growth in Amazonia. In fact, the ~9.39 million hectares of forest and *cerrado* lost to low-revenue land uses is an area twice as large as the total deforestation area of ~45,000 km^2^ estimated by INPE for the entire Legal Amazonian region between 2007 and 2011.

INCRA’s key mitigating assertion that deforestation within settlement areas occurred prior to the arrival of new settlers can be unambiguously dismissed, as even official data from governmental sources clearly show that agrarian reform areas tend to lose nearly one third of their remaining forest cover within 10 years of settlement occupation. This can be seen in a clear triangular envelope representing higher variances in annual deforestation when settlers arrive at increasingly forested areas (Fig 9b), for the simple reason that absolute deforestation rates are necessarily lower in areas that had already lost most of their forest cover. During the first post-settlement decade, the incidence of fires also increased dramatically. Fires in Amazonia are spatially correlated with recent deforestation [75] and the timing and spatial distribution of deforestation and fires in our analysis provides irrefutable chronological and spatially explicit evidence of agropastoral conversion both inside and immediately outside agrarian settlements 10 years after settlement occupation. Not only settlement areas retain less forest than the municipal counties in which they are established, but agrarian policy maximizes those impacts by selecting primarily forested regions, side-stepping standard environmental licensing procedures, and allocating small family plots that will unavoidably demand higher land-use intensity to achieve minimum living standards.

Contrary to the common-sense notion that Amazonian deforestation is merely a product of rampaging capitalist development unleashed by free market forces, it is primarily a governance problem that is deliberately designed and deployed by government, and funded by Brazilian tax-payers. Disagreement among different federal agencies is at the root of current deforestation trends across Amazonia, and the national treasury continues to pour millions of dollars in subsidizing deforestation, rather than in truly sustainable development options. Moreover, this form of government expenditure will hardly achieve the eradication of rural poverty, due to the economic inefficiencies of most agrarian settlements and the boom-and-bust patterns of development typical of the Amazonian frontier [76, 77], in which per capita living standards show an ephemeral improvement with deforestation, followed by a rapid decline in the aftermath of liquidation of the natural forest resource capital.

As far as we can foresee, the Brazilian agrarian reform program will continue to bestow land to the poor to reduce Brazil’s severely skewed farmland distribution. However, the colossal environmental costs and low net social benefits of this program should be mitigated by preventive measures including (1) preferentially allocating settlements to the 30 million hectares of degraded low-productivity pastures that have become available across the region [78], (2) avoiding frontier expansion into forested areas, (3) appropriate landscape-scale design of settlements to preclude conflicts with legally protected forest reserves and indigenous lands, and (4) enforcement of environmental licensing protocols. Finally, ‘best’ land-use practices in resettled areas should be encouraged and enforced. In sum, any prospects of conservation and sustainable development in Legal Amazonia requires that law compliance and public sector accountability be enforced, and that policy contradictions between competing government agencies be significantly minimized.
